# Xylose metabolism in the pig

**DOI:** 10.1371/journal.pone.0205913

**Published:** 2018-10-25

**Authors:** Nichole F. Huntley, John F. Patience

**Affiliations:** Department of Animal Science, Iowa State University, Ames, IA, United States of America; University of Illinois, UNITED STATES

## Abstract

It is important to understand if, and to what extent, the pig can utilize xylose as an energy source if xylanase releases free xylose in the small intestine. The experimental objectives were to determine the effects of industry-relevant dietary xylose concentrations and adaptation time on xylose retention efficiency and metabolism, diet digestibility and energy value, nitrogen balance, and hindgut fermentation. Forty-eight pigs were housed in metabolism crates and randomly assigned to one of four treatments with increasing D-xylose levels (n = 12/treatment) in 2 replications of a 22-d experiment with 3 collection periods. The control diet was xylose-free (0%), to which either 2, 4, or 8% D-xylose was added. Adaptation effects were assessed during three fecal and urine collection periods: d 5–7, 12–14, and 19–21. On d 22, pigs from the 0 and 8% treatments were euthanized; cecal and colon digesta were collected. Dietary xylose did not affect the total tract digestibility of dry matter, gross energy, or crude protein (*P*>0.10). Digesta short chain fatty acids concentrations and molar proportions and cecal pH were not different (*P*>0.10). This experiment utilized a targeted metabolomics approach to characterize and quantify urine xylose and metabolite excretion. Xylose retention decreased from 60% to 47% to 41% when pigs were fed diets containing 2, 4, or 8% xylose, respectively. In the 4 and 8% treatments, xylose retention was greater in the 2^nd^ and 3^rd^ collection periods compared to the 1^st^. A comprehensive pathway for xylose metabolism was proposed and D-threitol was confirmed as the major urinary metabolite of xylose. In conclusion, pigs can metabolize xylose, but with considerably lower efficiency than glucose, and may be able to adapt with time to utilize xylose more efficiently.

## Introduction

Xylose is a primary component of arabinoxylan in the hemicellulose fraction of carbohydrates in swine diets. Xylose is a potential energy source within the fiber structure of arabinoxylan, which is unavailable to the pig as a monosaccharide but can contribute some energy through fermentation. Xylanase hydrolyzes the β-linked xylose backbone of arabinoxylan and releases a mixture of oligosaccharides, disaccharides, and monomeric pentose sugars [1–3] such as xylose. Xylose can be absorbed by the small intestine and thus could potentially contribute energy to the pig; yet little information is available on xylose metabolism in pigs and mammals in general [4]. It is important to understand the energy available from xylose because nutritionists formulate diets to precisely balance the concentrations of energy and nutrients. Therefore, to effectively utilize xylanase to improve pig performance and efficiency when fed high fiber diets, we must understand how xylanase supplementation impacts dietary energy availability through the release of free xylose.

Most research on xylose metabolism in pigs has been conducted utilizing diets with xylose concentrations ranging from 5–40% [4]. However, typical growing pig diets will contain only 3–5% xylose as arabinoxylan, and not all of this would be released as free xylose in the small intestine. Additionally, most studies have allowed only 4–7 d of adaptation to treatment diets, so it is yet to be determined if pigs develop increased capacity for xylose metabolism as the length of exposure increases. Therefore, the objectives of this experiment were to determine the effects of industry-relevant dietary xylose concentrations and adaptation time on xylose retention efficiency and metabolism, diet digestibility and energy value, nitrogen (N) balance, and hindgut fermentation, and to identify urinary metabolites and propose a metabolic pathway of xylose using a targeted metabolomics approach.

## Materials and methods

All experimental procedures adhered to guidelines for the ethical and humane use of animals for research and were approved by the Iowa State University Institutional Animal Care and Use Committee (2-17-8452-S).

### Animals, housing, and experimental design

The experiment was conducted at the Iowa State University Swine Nutrition Farm (Ames, IA) using 48 crossbred gilts (Genetiporc 6.0 × Genetiporc F25, PIC, Inc., Hendersonville, TN) with an initial body weight (BW) of 28.3 ± 1.5 kg in two replications using 24 pigs each.

Prior to the experiment, pigs were housed individually for 4 d in pens (1.8 × 1.9 m) with slatted concrete floors for adaptation to individual housing and to the basal diet and feeding schedule. At the start of the trial, pigs were randomly allotted to one of four dietary treatments and were individually housed in metabolism crates (0.7 × 1.5 m) for the 22-d experiment. The metabolism crates allowed for separate collection of feces and urine and were equipped with a slatted floor, feeder, and nipple waterer that was attached to a modified water container to allow accurate measurement of water intake. Pigs were weighed on d 0 and 22. On the last day of the experiment (d 22) a total of 24 pigs, 12 from each replication (six each from the 0% and 8% treatments), were euthanized for tissue collection via captive bolt stunning and exsanguination.

### Diets and feeding

Four dietary treatments with increasing levels of D-xylose were evaluated (Table 1). The basal control diet was xylose-free (0%) and the following three treatments consisted of either 2%, 4%, or 8% D-xylose (DuPont Nutrition & Health, Copenhagen, Denmark) added to the basal diet at the expense of corn starch. All diets were formulated to meet or exceed NRC [5] (2012) requirements for growing pigs (Table 2) and were manufactured in mash form. The same batch of feed was used for both replications. During diet mixing, representative diet samples were obtained by homogenizing 10 subsamples; these were stored at -20°C for future analysis. Titanium dioxide (TiO_2_) was included in the diet as an indigestible marker to determine nutrient digestibility. Four days prior to initiation of the experiment, all pigs were offered the 0% diet. On d 0, pigs were transitioned to their assigned dietary treatments at 4% of the average initial BW. The daily feed allotment was split into two daily feedings at 0800 and 1600 h. Any orts remaining after 1 h were collected and weighed. Pigs in both replicates received the same amount of feed. Pigs had ad libitum access to water throughout the entire trial.

**Table 1:** Diet ingredient composition, as-fed.

	Dietary treatment, % xylose inclusion			
Item, %	0	2	4	8
Corn starch	57.00	55.00	53.00	49.00
Sucrose	8.60	8.60	8.60	8.60
Cellulose	9.00	9.00	9.00	9.00
Casein	5.50	5.50	5.50	5.50
Bovine plasma	5.00	5.00	5.00	5.00
Fish meal	4.50	4.50	4.50	4.50
Skim milk powder	3.00	3.00	3.00	3.00
Whey powder	3.00	3.00	3.00	3.00
Soybean oil	1.00	1.00	1.00	1.00
D-Xylose^1^	0.00	2.00	4.00	8.00
Limestone	0.90	0.90	0.90	0.90
Monocalcium phosphate	0.35	0.35	0.35	0.35
Choline chloride	0.05	0.05	0.05	0.05
Potassium carbonate	0.50	0.50	0.50	0.50
Magnesium oxide	0.07	0.07	0.07	0.07
Lysine HCl	0.05	0.05	0.05	0.05
DL-Methionine	0.10	0.10	0.10	0.10
L-Threonine	0.03	0.03	0.03	0.03
Vitamin premix^2^	0.25	0.25	0.25	0.25
Trace mineral premix^3^	0.20	0.20	0.20	0.20
Salt	0.50	0.50	0.50	0.50
Titanium dioxide	0.40	0.40	0.40	0.40

^1^DuPont Nutrition & Health, Copenhagen, Denmark

^2^Provided 6,614 IU vitamin A, 827 IU vitamin D, 26 IU vitamin E, 2.6 mg vitamin K, 29.8 mg niacin, 16.5 mg pantothenic acid, 5.0 mg riboflavin, and 0.023 mg vitamin B12 per kg of diet.

^3^Provided 165 mg Zn (zinc sulfate), 165 mg Fe (iron sulfate), 39 mg Mn (manganese sulfate), 17 mg Cu (copper sulfate), 0.3 mg I (calcium iodate), and 0.3 mg Se (sodium selenite) per kg of diet.

**Table 2:** Formulated and analyzed diet energy and nutrient composition, as-fed.

	Dietary treatment, % xylose inclusion			
Item	0	2	4	8
Formulated composition				
SID amino acid, %				
Lys	1.02	1.02	1.02	1.02
Met	0.36	0.36	0.36	0.36
Total sulfur AA	0.56	0.56	0.56	0.56
Thr	0.61	0.61	0.61	0.61
Trp	0.18	0.18	0.18	0.18
Ca, %	0.66	0.66	0.66	0.66
STTD P, %	0.33	0.33	0.33	0.33
Analyzed composition				
DM, %	94.02	94.90	95.26	95.58
GE, MJ/kg	16.41	16.25	16.53	16.55
CP, %	14.05	14.03	14.07	14.03
aEE, %	1.62	1.57	1.76	1.52
NDF, %	10.31	10.40	10.49	10.56
ADF, %	7.62	7.71	7.62	8.00
Xylose, %	0.00	1.99	4.02	8.02

Pigs (n = 12/treatment) were housed individually in metabolism crates and fed diets containing either 0, 2, 4, or 8% D-xylose at 4% of BW and fecal samples were collected during 3 different periods representing increasing adaptation time to treatment diets.

SID, standardized ileal digestible; AA, amino acids; STTD, standardized total tract digestible; GE, gross energy; CP, crude protein; aEE, acid hydrolyzed diethyl ether extract; NDF, neutral detergent fiber; ADF, acid detergent fiber.

### Sample collection

The effect of adaptation time was assessed by utilizing three fecal and urine collection periods throughout the 22-d experiment. Collection periods 1 (d 5–7; C1), 2 (d 12–14; C2) and 3 (d 19–21; C3) allowed pigs to adapted to the treatment diets for 4, 11, and 18 d prior to each collection, respectively.

Each collection period lasted 72 h and total urine and fresh fecal grab samples were collected at 0830 and 1630 h each day. Fecal samples were compiled and homogenized within each collection period and immediately stored at -20°C. Urine was collected quantitatively into 4-litre bottles containing 6 *M*-HCl to ensure that the pH was maintained below 2.0 to minimize nitrogen (N) volatilization. Total urine output was weighed and stored at -20°C. After each collection period was completed, urine was thawed, homogenized, strained through glass wool, subsampled, and again stored -20°C for later analysis. Urine specific gravity was measured by weighing a homogenized subsample of 500 ± 1 ml.

Water intake was also measured during each collection period. A 12-litre plastic container was modified to attach to the nipple waterers already installed in the metabolism crates (Hog Slat, #HSNA-50, Newton Grove, NC) and the internal screens were removed. The containers were placed on top of each crate to allow water to be gravity-fed into each waterer. The containers were emptied at the beginning of each collection period and the volume of water added throughout the collection period was recorded. At the end of the 72-h period the volume of water remaining was recorded to calculate water disappearance. Correspondingly, at the beginning of each collection period, trays were placed underneath the front of each crate to collect and measure the volume of wasted water. Water intake was then calculated by subtracting water waste from water disappearance during each collection period.

On d 22, 24 total pigs (12 pigs from the 0% treatment and 12 pigs from the 8% treatment) were euthanized 60 ± 3 min after feeding. The abdomen of the carcass was opened and, to minimize digesta movement, the gastrointestinal tract was clamped at the stomach/duodenum sphincter, the ileum/cecum junction, the cecum/large intestine junction, and at the rectum. The intestines were then removed from just below the stomach to near the anus. Digesta pH was measured at the cecum and mid-colon, and digesta from both sections was collected, and immediately frozen. Digesta samples were stored on dry ice for transportation to the lab and stored at -20°C until analysis.

### Analytical methods and calculations

All diet, orts, and fecal samples were dried at 60°C to a constant weight and were ground to a particle size of 1 mm. Urine subsamples were thawed, mixed, and filtered through Whatman 41 filter paper (GE Healthcare Life Sciences, Chicago, IL, USA) prior to analysis. Urine, diet, and fecal samples were analyzed in duplicate for N (method 990.03 [6]; TruMac; LECO Corp., St. Joseph, MI, USA). Standard calibration was performed using EDTA (9.56% N) which was determined to contain 9.55 ± 0.01% N. Crude protein (CP) was calculated as N x 6.25.

Diet and fecal samples were analyzed in duplicate for dry matter (DM; method 930.15), TiO_2_ and gross energy (GE). Titanium dioxide was determine according to the colorimetric method of Leone (1973) [7]. For urine energy determination, 3 ml of urine was added to 0.5 g of dried cellulose (SolkaFloc, International Fiber Corporation, North Tonawanda, NY, USA), subsequently dried at 50°C for 72 h, and urine plus cellulose dried samples were analyzed in duplicate for GE. Gross energy was determined using a bomb calorimeter (model 6200; Parr Instrument Co., Moline, IL). Benzoic acid (26.43 MJ/kg; Parr Instrument Co.) was used as the standard for calibration and was determined to contain 26.43 ± 0.01 MJ/kg. Urinary energy was calculated from the difference of energy determined in cellulose and the energy determined in the samples containing both urine and cellulose.

Apparent total tract digestibility (ATTD) of dry matter, GE, and CP were calculated according to the equation of Oresanya et al. [8], as 100 –{100 × [concentration (g) of TiO_2_ in diet × concentration (g) of DM, GE, or CP in feces]/[concentration (g) of TiO_2_ in feces × concentration (g) of DM, GE, or CP in diet]}.

Diets and a representative portion of the fecal and colon digesta samples (n = 24) were also analyzed for D-xylose concentration using a commercially available kit (Megazyme, Wicklow, Ireland). To prepare diet samples, they were ground to 0.5 mm particle size and acid hydrolyzed according to manufacturer’s instructions. Fecal and colon digesta samples used for D-xylose analysis were lyophilized, ground to 0.5 mm particle size, and clarified according to manufacturer’s instructions. In all fecal and colon digesta samples, the xylose concentration was determined to be < 1% (average = 0.24%), which agrees with previous literature that free xylose is nearly completely absorbed in the small intestine [9]. Therefore, the ATTD of xylose was not measured. Diets were also analyzed in duplicate for acid hydrolyzed ether extract (aEE; method 2003.06) and in triplicate for neutral and acid detergent fiber components (NDF [10] and ADF [11], respectively). The analyzed nutrient composition of the diets is presented in Table 2.

Quantitative metabolomics analysis was completed for all urine samples using tandem GC/MS. Filtered urine samples were vortexed, 10 μl was transferred to a 2-ml glass vial, and 50 μl of ribitol (1 mg/ml in HPLC grade water) was added to each sample as an internal standard. The samples were then concentrated to complete dryness in a SpeedVac concentrator (Savant, Thermo Fisher Scientific Inc., Waltham, MA). The dried samples were treated with methoxamine (20 mg/ml methoxamine HCl in dry pyridine) followed by sonication at ambient temperature for 30 min and incubation at 60°C for 60 min. The samples were then derivatized with the addition of *N*,*O*-bis(trimethylsilyl)-trifluoroacetamide with 1% trimethylchlorosilane and incubated at 60°C for 30 min.

Analysis was performed on an Agilent 19091s-433 GC/MS system equipped with a HP-5ms chromatographic column (30 m × 250 μm × 0.25 μm; Santa Clara, CA). The injection temperature set to 280°C and the injected volume was 1 μl with a 1:10 split. The helium flow rate was 1 ml/min. The column temperature was initially set at 90°C for 2 min and then increased to 320°C at a rate of 15°C/min where it was held for 3 minutes. Mass spectral analysis was performed in the scan-total ion monitoring mode after electron ionization. The MS quadrupole temperature was set to 150°C and the ion source temperature was set to 230°C. The mass data were collected in the range from m/z 40 to m/z 800. The acceleration voltage was turned on after a solvent delay of 7 min.

The AMDIS software (National Institute of Standards and Technology (NIST), Gaithersburg, MD) was used for peak quantification. A hydrocarbon ladder was used for retention index calculation and standards of D-threitol (Sigma-Aldrich, Saint Louis, MO), D-xylose, D-erythritol, xylitol, D-xylulose, and D-xylonic acid (Supelco, Bellefonte, PA) were used for compound identification. Additional compounds were identified using NIST14 Mass Spectral Library (version 2.2) based on mass spectral matching. The integrated peak areas of multiple derivative peaks belonging to the same compound were summed and considered as a single compound. Concentrations of identified compounds were calculated using the ratio of the peak area of each compound to the internal standard; and D-xylose, D-threitol, D-erythritol, xylitol, D-xylulose, and D-xylonic acid were quantified based on a standard curve regression.

Urine GE (kJ/l) contributed from xylose, threitol, xylitol, xylonic acid, and xylulose were calculated as the concentration of the compound (g/l) × GE of the compound (kJ/g). Urine GE contributed by N-containing compounds was estimated by applying an assumption that all urinary N was in the form of urea and was calculated as the urine N concentration (g/l) × 10.54 (kJ /g, the GE of urea). Urine GE not allocated to xylose, metabolites, or N-containing compounds was calculated as urine GE (kJ /l)–[urine GE from xylose + threitol + xylitol + xylonic acid + xylulose + N-containing compounds (kJ /l)].

Digesta samples from the cecum and colon were analyzed for short chain fatty acid (SCFA) concentrations. One gram of sample was diluted with 2.5 ml ddH_2_O and mixed with 0.2 ml of 25% metaphosphoric acid and 0.1 ml of isocaproic acid (48.3 m*M;* Sigma-Aldrich, Saint Louis, MO) as an internal standard. A standard curve was generated using five concentrations of acetate, propionate, butyrate, valerate, isobutyrate, and isovalerate (Sigma-Aldrich, Saint Louis, MO). The internal standard (0.1 ml) was added to each set of standards. Samples and standards were centrifuged, and the supernatant was transferred to a glass vial for analysis. Gas chromatography (3800 Varian GC, Agilent Technologies, Santa Clara, CA) was performed using a 15 m × 0.25 mm × 0.25 μm fused silica capillary column (Supelco, Bellefonte, PA) with flame ionization detection. Helium was used as the carrier gas at the flow rate of 1 ml/min. The injector temperature was 250°C and the oven temperature held at 143°C until increasing to 200°C for 6 min. Concentrations of acetate, propionate, butyrate, valerate, isobutyrate, and isovalerate were calculated using the ratio of the peak area of each compound to the internal standard and standard curve regression. Molar proportions of SCFA were calculated as the individual SCFA concentration (m*M*) / total SCFA concentration (m*M*).

### Statistical analyses

Data were analyzed as a three by four factorial design with pig as the experimental unit. The UNIVARIATE procedure of the Statistical Analysis Systems package version 9.4 (SAS Institute, Cary, NC, USA) was used to verify normality of residuals and homogeneity of variances and statistical outliers (> 3 standard deviations away from the mean) were removed. Water intake, urine output, and digesta SCFA molar proportions data were log transformed, and SCFA concentration data were reciprocal transformed to achieve a normal distribution. The statistical models for all SCFA data were fit with separate variances for each of the two treatments.

The fixed effects of dietary treatment (Trt), collection period (Col), and their interaction (Trt × Col), and the random effect of replication were analyzed using the MIXED procedure of SAS. Data were analyzed as repeated measures with pig as the subject and an unstructured covariance structure was applied. For data collected only at one time period (ie. growth performance and cecal and colon pH and SCFA), the fixed effect of dietary treatment was analyzed with replication as a random effect.

Differences due to dietary treatment, collection period, and their interaction were determined using ANOVA and means were separated using the least square means statement and the PDIFF option. Linear and quadratic polynomial contrasts with coefficients adjusted for unequal treatment spacing were used to determine the response to dietary xylose concentration. Differences were considered significant if *P* was ≤ 0.05 and a trend if *P* was > 0.05 and ≤ 0.10.

Urine metabolomics data were analyzed as grams of compound excreted per day to account for differences in urine volume. Multivariate analysis of log-transformed and range-scaled data was performed using MetaboAnalyst 4.0 [12] including repeated measures two-way ANOVA for the effects of dietary treatment and collection period, heat map generation, and the impact of dietary xylose concentration on metabolic pathways and metabolite set enrichment.

## Results

### Growth performance and water balance

Pigs were monitored for health twice per day and all pigs remained healthy throughout the entire trial. Initial BW and d 22 BW did not differ (Trt *P* ≥ 0.6659; Table 3) and pigs on all dietary treatments had similar average daily gain (ADG) and feed efficiency (Trt *P* ≥ 0.1653; Table 3). Water intake increased in a linear fashion as dietary xylose concentration increased; in turn, this resulted in a linear increase in urine output (Table 4; Trt *P* < 0.01). However, urine specific gravity did not differ among treatments (Table 4). A tendency for differences among dietary treatments (*P* = 0.0656) and a significant collection period effect (*P* = 0.0087) were detected for urine specific gravity, but these were likely due to the very small standard error of the mean (SEM; 0.01). In any event, this magnitude of differences would not be biologically relevant. Collection period significantly impacted water balance as well as water waste, with pigs wasting less water during C2 and C3 compared to C1 (*P* = 0.0001; Table 4).

**Table 3:** Effect of dietary xylose concentration on pig growth performance.

	Dietary treatment, % xylose inclusion					
Item	0	2	4	8	SEM	*P*-value
BW, kg						
d 0	28.18	28.27	28.38	28.20	0.46	0.9895
d 22	40.90	40.23	40.75	40.28	0.46	0.6659
ADG, g/d	606	570	589	576	20	0.1653
Gain: feed	0.53	0.50	0.52	0.51	0.02	0.1660

Pigs (n = 12/treatment) were housed individually in metabolism crates and fed diets containing either 0, 2, 4, or 8% D-xylose at 4% of BW for 22 d.

BW, body weight; ADG, average daily gain

**Table 4:** Effect of dietary xylose concentration and collection period on water balance.

	Dietary treatment, % xylose inclusion						Collection period					T×C^1^
Item	0	2	4	8	SEM	*P*-value	d 5–7	d 12–14	d 19–21	SEM	*P*-value	*P*-value
Water waste, ml/d	853	796	1024	1265	321	0.3633	1404^x^	712^y^	837^y^	294	0.0001	0.4096
Water intake^‡^, ml/d	2447^b^	2532^b^	2762^a^^b^	3017^a^	222	0.0424	3025^x^	2551^y^	2493^y^	176	<0.0001	0.4352
Urine output^‡^, ml/d	984^c^	1159^b^^c^	1286^a^^b^	1566^a^	129	0.0032	1465^x^	1196^y^	1085^z^	74	<0.0001	0.7898
Urine specific gravity	1.034	1.031	1.025	1.029	0.009	0.0656	1.029^x^^y^	1.028^y^	1.032^x^	0.009	0.0087	0.2528

Pigs (n = 12/treatment) were housed individually in metabolism crates and fed diets containing either 0, 2, 4, or 8% D-xylose at 4% of BW. Water intake and urine output were measured during 3 different collection periods representing increasing adaptation time to treatment diets.

^a-c^Treatment means without a common superscript differ (*P* ≤ 0.05)

^x-z^Collection period means without a common superscript differ (*P* ≤ 0.05)

^1^Dietary treatment × collection period interaction (T×C)

^‡^Treatment linear contrast is significant at *P* < 0.01

### Diet digestibility and energy value and nitrogen balance

The ATTD of DM, GE, and CP did not differ among treatments (Trt *P* ≥ 0.4074; Table 5), but the ATTD was significantly impacted by collection period (Col *P* ≤ 0.0014). There were significant interactions between treatment and collection period for DM and GE ATTD (Trt × Col *P* ≤ 0.0003) and a trend for interaction for CP ATTD (Trt × Col *P* = 0.0858; Table 5). Small differences in GE ATTD resulted in a tendency for an interaction of treatment and collection period on diet digestible energy (DE, MJ/kg; Table 5). Dietary treatment affected diet DE with pigs on the 2% xylose treatment having lower DE value compared to all other treatments (14.91 MJ/kg vs. an average of 15.12 MJ/kg; Trt *P* = 0.0004). However, the SEM for all digestibility variables was relatively low (≤ 0.50) which likely resulted in detection of the interaction.

**Table 5:** Effect of dietary xylose concentration and collection period on diet digestibility and energy value and on nitrogen balance.

Collection period, d	5–7				12–14				19–21					
Dietary treatment, % xylose inclusion	0	2	4	8	0	2	4	8	0	2	4	8	Pooled SEM	T×C^1^ *P*-value
ATTD, %														
DM^†^	87.82^d^^e^	88.49^a^^b^^c^^d^	88.39^a^^b^^c^^d^^e^	88.12^b^^c^^d^^e^	88.14^c^^d^^e^	89.10^a^	88.89^a^^b^	88.58^a^^b^^c^^d^	88.70^a^^b^^c^	87.56^e^	88.80^a^^b^^c^	88.06^c^^d^^e^	0.30	0.0003
GE^†^	88.98^e^^f^	89.45^b^^c^^e^	89.43^a^^b^^c^^e^^f^	89.33^b^^c^^e^^f^	89.39^c^^e^	90.18^a^	90.05^a^^b^	89.69^a^^b^^c^^e^	90.00^a^^b^^d^	88.62^f^	90.01^a^^b^^c^	89.19^c^^d^^e^^f^	0.29	<0.0001
CP^†^	90.86	91.16	91.29	90.74	91.96	92.18	91.78	91.64	92.62	91.95	92.57	91.59	0.50	0.0858
Urine GE*^‡^^†^, kJ/l	715	886	922	1209	889	1009	1144	1306	939	1043	1256	1372	70	0.5803
Diet energy value, MJ/kg, as-fed														
DE*^#^^†^	14.91	14.82	15.09	15.02	15.09	14.98	15.17	15.17	15.20	14.94	15.30	15.16	0.08	0.0857
ME*^‡^^†^	13.81^b^^c^	13.54^d^^e^	13.50^e^	13.00^g^	13.95^b^	13.74^b^^c^	13.67^c^^d^^e^	13.27^f^	14.09^a^	13.54^e^	13.72^c^^d^	13.14^f^^g^	0.08	0.0116
Urine N*^‡^^†^, %	0.28	0.31	0.20	0.21	0.43	0.39	0.33	0.29	0.50	0.44	0.41	0.34	0.03	0.0962
Nitrogen balance														
Retained, % of intake	76.70	75.08	78.72	75.98	76.32	75.97	76.03	75.90	76.25	75.85	75.69	73.19	1.62	0.1497
N in feces^†^, % of excreted N	59.98	63.08	58.52	61.03	65.30	66.25	64.86	66.02	67.97	66.12	69.22	69.18	2.03	0.2949
N in urine^†^, % of excreted N	40.02	36.92	41.48	38.97	34.70	33.75	35.14	33.98	32.03	33.88	30.78	30.82	2.03	0.2949

Pigs (n = 12/treatment) were housed individually in metabolism crates and fed diets containing either 0, 2, 4, or 8% D-xylose at 4% of BW. Feces and urine were collected during 3 different periods representing increasing adaptation time to treatment diets.

ATTD, apparent total tract digestibility; GE, gross energy; CP, crude protein; ME, metabolizable energy; N, nitrogen

^a-g^Within a row, means without a common superscript differ (*P* ≤ 0.05)

^1^Dietary treatment × collection period interaction (T×C)

*Treatment is significant at *P* < 0.01

^#^Treatment linear contrast is significant at *P* < 0.05

^‡^Treatment linear contrast is significant at *P* < 0.01

^†^Collection period is significant at *P* < 0.01

Urine N (%) decreased linearly with increasing dietary xylose concentration (Trt *P* = 0.009, linear *P* = 0.009; Table 5) but increased across collection periods as pigs had longer adaptation time to the diets (Col *P* < 0.0001). There tended to be an interaction between treatment and collection period for urine N with concentrations increasing across collection periods for all treatments, but to a lower magnitude in the 8% xylose treatment (Table 5). Nitrogen balance (retained N and the percent of N excreted in the urine or feces) was not affected by dietary treatment (Trt *P* ≥ 0.5738; Table 5). As pigs had longer adaptation time, the proportion of N excreted in the feces increased (Col *P* < 0.0001) while the proportion excreted in the urine decreased (Col *P* < 0.0001).

Urine energy concentration (kJ/l) linearly increased with increasing dietary xylose concentration (Trt *P* < 0.0001, linear *P* < 0.0001) and increased across collection periods as pigs had longer adaptation time to the treatment diets (Col *P* < 0.0001). This resulted in a significant treatment by collection period interaction for diet metabolizable energy (ME, MJ/kg) value (Table 5).

### Digesta short chain fatty acids

Short chain fatty acid concentrations (m*M*) and molar proportions in the cecum and colon did not differ between the 0 and 8% treatments (Trt *P* ≥ 0.2534; Table 6). The pH of the cecal digesta did not differ (Trt *P* = 0.3845) but colon digesta in pigs fed 8% xylose had a lower pH (Trt *P* = 0.0249; Table 6).

**Table 6:** Effect of dietary xylose inclusion on cecum and colon digesta pH and SCFA concentration and molar proportions.

	Dietary treatment, % xylose inclusion				
	0	SEM	8	SEM	*P*-value
Digesta pH					
Cecum	6.64	0.11	6.54	0.11	0.3845
Colon	6.52	0.10	6.17	0.10	0.0249
Cecal digesta SCFA concentration, mmol/g					
Acetate	22.55	1.76	22.55	1.11	0.8394
Propionate	8.82	0.70	8.90	0.62	0.9281
Butyrate	2.13	0.16	2.46	0.29	0.4230
Valerate	0.87	0.06	0.91	0.09	0.7377
Isobutyrate	0.68	0.04	0.68	0.04	0.8756
Isovalerate	1.14	0.09	1.10	0.05	0.6613
Total	36.20	2.42	37.03	1.62	0.5341
Cecal digesta SCFA molar proportion, %					
Acetate	61.97	1.78	62.02	1.40	0.9819
Propionate	24.41	1.24	23.97	1.18	0.7782
Butyrate	6.00	0.65	6.59	0.78	0.4075
Valerate	2.43	0.12	2.51	0.24	0.9100
Isobutyrate	1.96	0.16	1.89	0.15	0.7435
Isovalerate	3.23	0.27	3.02	0.14	0.4632
Colon digesta SCFA concentration, mmol/g					
Acetate	22.20	2.09	22.12	2.19	0.9731
Propionate	8.39	0.73	8.91	0.79	0.7775
Butyrate	3.65	0.41	3.60	0.34	0.9181
Valerate	1.00	0.11	1.01	0.11	0.8466
Isobutyrate	0.72	0.06	0.64	0.06	0.3509
Isovalerate	1.38	0.09	1.22	0.09	0.2534
Total	37.34	1.97	37.51	2.11	0.9474
Colon digesta SCFA molar proportion, %					
Acetate	59.06	2.73	58.55	2.97	0.8741
Propionate	22.57	1.68	23.90	2.07	0.6321
Butyrate	9.85	1.34	9.62	1.18	0.9392
Valerate	2.70	0.29	2.73	0.31	0.9706
Isobutyrate	2.04	0.23	1.80	0.24	0.4379
Isovalerate	3.79	0.33	3.40	0.40	0.4664

Pigs (n = 12/treatment) were housed individually in metabolism crates and fed diets containing either 0 or 8% D-xylose at 4% of BW for 22 d.

### Urine metabolomics

**Fig 1:**
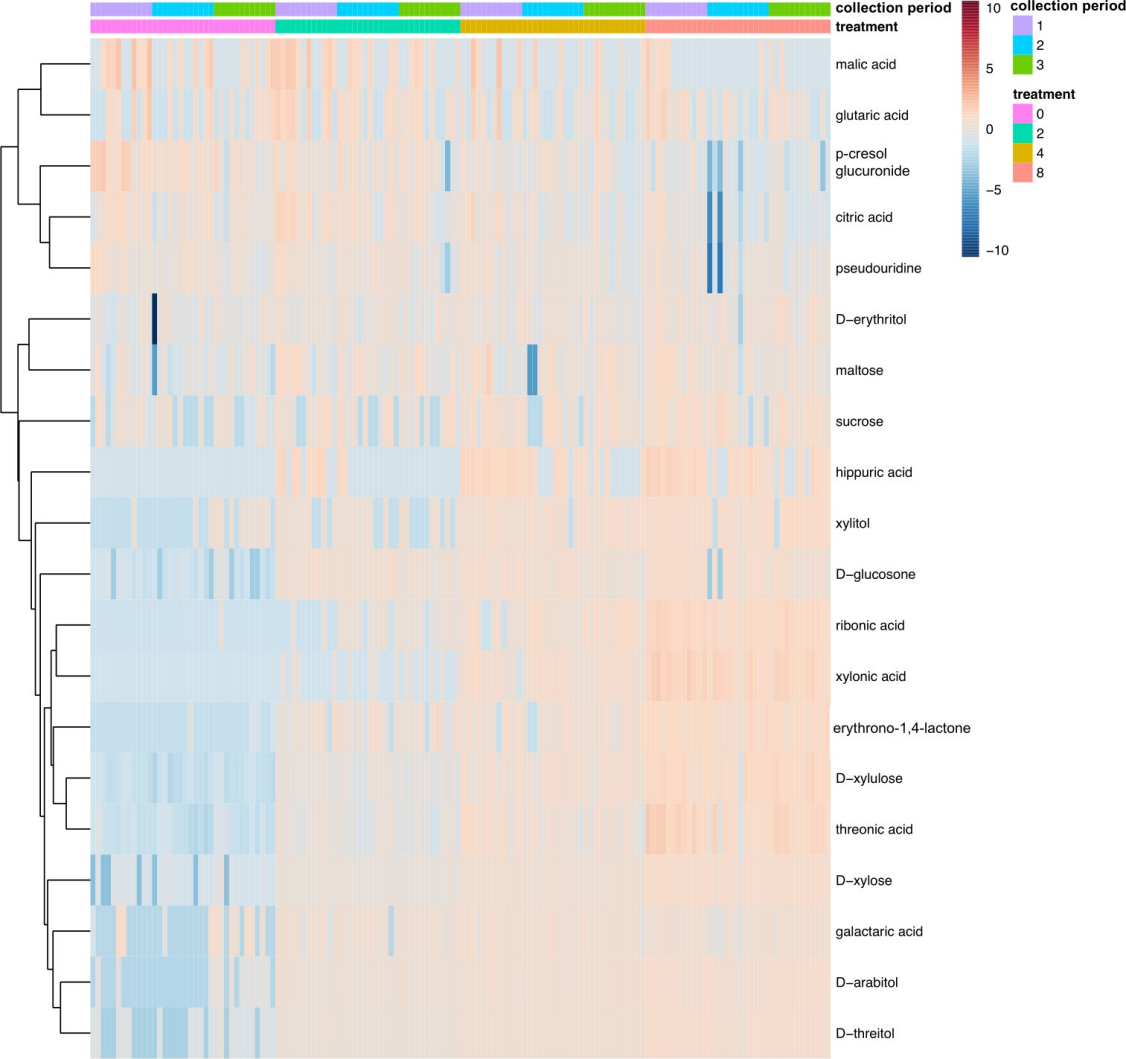
Two-way heat map visualization and hierarchical clustering of urine metabolite distribution. Pigs (n = 12/treatment) were fed diets containing either 0, 2, 4, or 8% D-xylose and 3 different collection periods were utilized to represent increasing adaptation time to treatment diets. The rows display the metabolites and the columns represent individual samples. Relative urinary metabolite excretion (g/d, log transformed) is represented in the heat map by colors, which correspond to the magnitude of difference when compared with the average value for the metabolite. Only metabolites significantly (adjusted *P* ≤ 0.05) impacted by treatment (% dietary xylose inclusion; 0, 2, 4, or 8%), collection period (1 = d 5–7, 2 = d 12–14, 3 = d 19–21), or their interaction are displayed. Significance was determined using two-way ANOVA.

Fig 1 displays the distribution patterns of compounds identified in urine from pigs fed increasing dietary xylose concentrations and among three collection periods representing increasing adaptation time to treatment diets. The Euclidean distance measure and the unweighted paired group average linkage technique for hierarchical clustering were applied. In total, 22 compounds were identified in the urine samples and 20 were determined to be differentially excreted due to dietary treatment, collection period, or their interaction (adjusted *P* < 0.05; Fig 1). All compounds were impacted by collection period, except glutaric acid and malic acid which were significantly affected only by treatment. Erythrono-1,4-lactone was only affected by the main effect of collection period. In general, increasing dietary xylose concentration increased the excretion of metabolites in the urine with the exceptions of malic acid, glutaric acid, pseudouridine, citric acid, and *p*-cresol glucuronide for which excretion decreased (Trt adjusted *P* < 0.05).

### Xylose metabolic pathway

**Fig 2:**
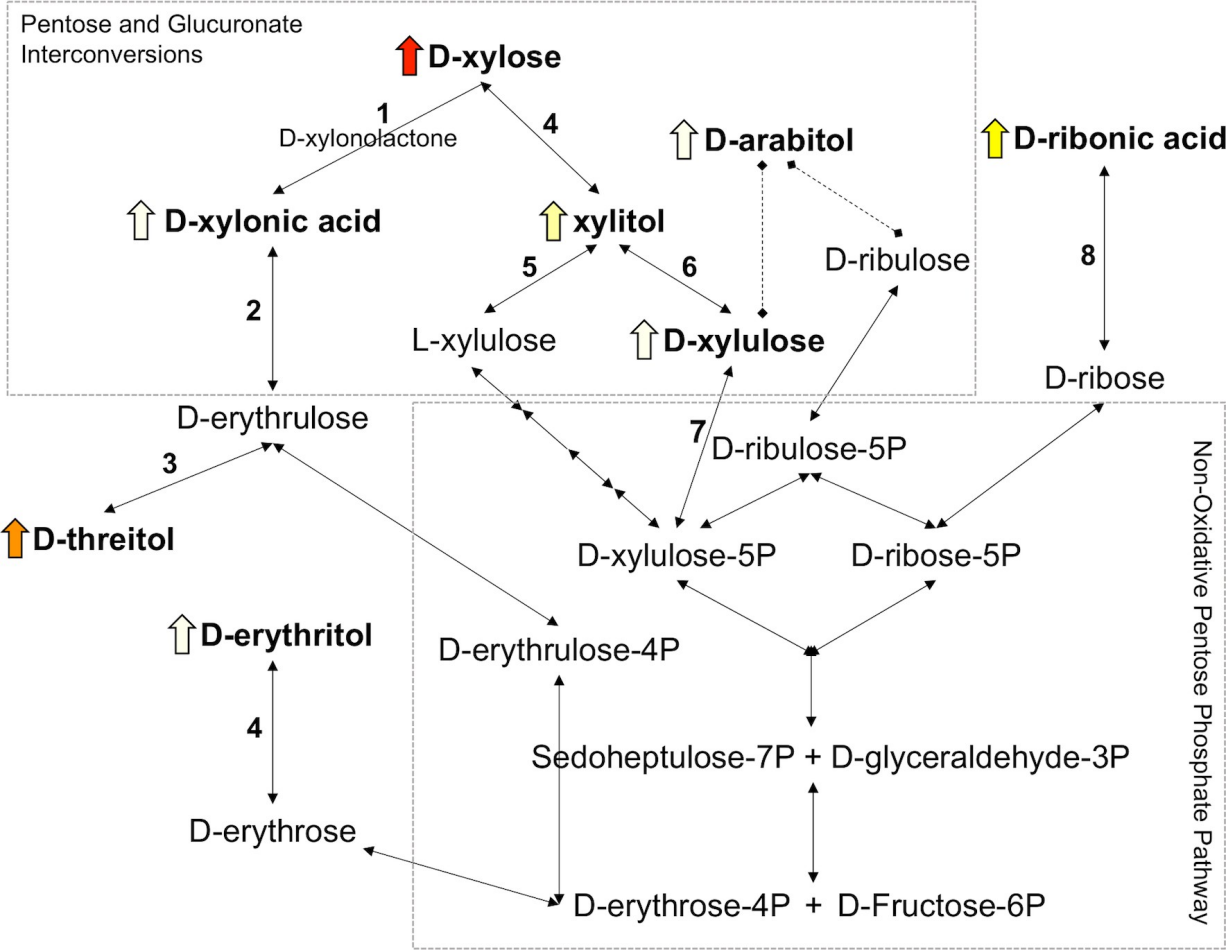
Proposed xylose metabolic pathway. Dietary D-xylose concentration (0, 2, 4, or 8%) linearly increased the urinary excretion of the compounds in bold font (*P* < 0.0001). Non-bolded compounds were not detected in urine samples. The color of the block arrow indicates average excretion amount of the compound in pigs from the 8% xylose treatment; red: 40 g/d, orange: 13 g/d, yellow: 0.3 g/d, white: < 0.2 g/d. Solid connecting arrows represent reactions confirmed in mammals and dashed connecting lines represent presumed reactions based on reactions occurring in microorganisms. Highlighted enzymes: 1) D-xylose 1-dehydrogenase (1.1.1.179), 2) L-gulonate 3-dehydrogenase (1.1.1.45), 3) D-erythrulose reductase (1.1.1.162), 4) aldose/aldehyde reductase (1.1.1.21), 5) L-xylulose reductase (1.1.1.10), 6) xylitol dehydrogenase (1.1.1.14), 7) xylulokinase (2.7.1.17), and 8) D-ribose dehydrogenase (1.1.1.115). The boxes indicate KEGG metabolic pathway classifications [36].

Metabolomic analysis was used to identify metabolic pathways impacted by xylose consumption and determine metabolite set enrichment. According to the pathway enrichment analysis, the pentose and glucuronate interconversions pathway was the most significantly impacted (Trt *P* < 0.001) and contained the largest proportion of identified compounds (4/22) that were enriched in the pathway. Based on this analysis, a review of the literature on xylose metabolism in pigs [13–24] and other mammals [25–35] where data specifically in pigs were not available, and validation using KEGG metabolic pathways [36] and BRENDA comprehensive enzyme database [37], a pathway for the metabolism of xylose is proposed (Fig 2). Clustering results (Fig 1) and interpretation of the xylose metabolic pathway (Fig 2) were used to determine that the most plausible direct metabolites of dietary xylose were considered to be D-threitol, xylitol, D-xylulose, and D-xylonic acid, in addition to xylose excreted in the urine unchanged. The excretion of all other identified compounds in the urine significantly affected by dietary xylose concentration were considered to have been indirectly affected as opposed to directly converted from dietary xylose.

### Xylose metabolite excretion

The excretion of xylose and its metabolites are presented in Table 7. Xylose was the most abundant compound identified in urine from pigs on the 2, 4, and 8% xylose treatments. Xylose excretion increased as xylose consumption increased (Trt *P* < 0.0001, linear *P* < 0.0001, quadratic *P* < 0.0001). In the 0 and 2% xylose treatments, xylose excretion was similar across collection periods, but in the 4 and 8% treatments, excretion was decreased in C2 and C3 compared to C1 (Col *P* < 0.0001). In the 4% treatment, xylose excretion was not different between C2 and C3 but in the 8% treatment, xylose excretion was greater in C3 compared to C2, but still less than C1 (Trt × Col *P* < 0.0001; Table 7). In pigs fed xylose-containing diets, the percent of consumed xylose excreted in the urine increased as dietary xylose concentration increased (Trt *P* < 0.0001, linear *P* < 0.0001; Table 8) but this proportion decreased after the first collection period (Col *P* < 0.0001). In the 8% treatment, the proportion was greater in C3 compared to C2 (Trt × Col *P* = 0.0026, Table 8).

**Table 7:** Effect of dietary xylose concentration and collection period on urine xylose and metabolite excretion.

Collection period, d	5–7				12–14				19–21					
Dietary treatment, % xylose inclusion	0	2	4	8	0	2	4	8	0	2	4	8	Pooled SEM	T×C^1^ *P*-value
Urinary excretion, g/d														
Xylose*^‡^^†^	0.51^g^	7.04^f^	21.59^d^	50.06^a^	0.49^g^	6.01^f^	16.25^e^	31.53^c^	0.83^g^	5.73^f^	16.06^e^	38.58^b^	1.28	< 0.0001
Threitol*^‡^^†^	0.12^f^	2.73^e^	6.53^d^	15.96^a^	0.08^f^	2.36^e^	5.61^d^	10.95^c^	0.27^f^	2.46^e^	6.01^d^	13.46^b^	0.45	0.0008
Xylitol*^‡^^†^	0.01^f^	0.07^e^	0.16^c^^d^	0.27^b^	0.01^f^	0.06^e^^f^	0.14^d^	0.23^b^	0.04^e^^f^	0.05^e^^f^	0.21^b^^c^	0.41^a^	0.02	0.0021
Xylulose*^‡^	0.02^e^	0.08^d^	0.12^c^	0.20^a^	0.02^e^	0.08^d^	0.13^c^	0.17^b^	0.02^e^	0.07^d^	0.13^c^	0.21^a^	0.007	0.0021
Xylonic acid^‡^^†^	0.000^c^^d^	0.001^c^^d^	0.009^c^^d^	0.059^a^	0.000^d^	0.001^d^^e^	0.009^c^	0.033^b^	0.000^d^	0.001^d^	0.008^c^^e^	0.035^b^	0.003	< 0.0001

Pigs (n = 12/treatment) were housed individually in metabolism crates and fed diets containing either 0, 2, 4, or 8% D-xylose at 4% of BW. Urine was collected during 3 different periods representing increasing adaptation time to treatment diets.

^a-g^Within a row, means without a common superscript differ (*P* ≤ 0.05)

^1^Dietary treatment × collection period interaction (T×C)

*Treatment is significant at *P* < 0.01

^‡^Treatment linear contrast is significant at *P* < 0.01

^†^Collection period is significant at *P* < 0.01

**Table 8:** Effect of dietary xylose concentration and collection period on urine energy concentration and urine sugar concentration and excretion.

Collection period, d	5–7			12–14			19–21				
Dietary treatment, % xylose inclusion	2	4	8	2	4	8	2	4	8	Pooled SEM	T×C^1^ *P*-value
Consumed xylose excreted in urine, %											
As xylose*^‡^^†^	31.09^c^^d^	47.17^b^	54.87^a^	26.53^c^^e^	35.50^c^	34.56^c^	25.32^e^	35.08^c^	42.29^b^	2.34	0.0026
As threitol*^‡^^†^	12.05	14.27	17.49	10.40	12.26	12.00	10.88	13.13	14.75	0.71	0.1037
As xylitol, xylulose, or xylonic acid*^#^	0.94^a^^b^^c^	0.89^a^^b^^c^	0.80^c^	0.92^a^^b^^c^	0.90^b^^c^	0.73^c^	0.86^a^^b^^c^^d^	1.03^a^	0.98^a^^b^	0.06	0.0180
Retained xylose*^‡^^†^, %	55.92^b^^c^	37.67^d^	26.84^e^	62.15^a^^b^	51.35^c^	52.77^c^	62.94^a^	50.77^c^	42.01^d^	2.85	0.0063

^a-e^Within a row, means without a common superscript differ (*P* ≤ 0.05)

^1^Dietary treatment × collection period interaction (T×C)

*Treatment is significant at *P* < 0.01

^‡^Treatment linear contrast is significant at *P* < 0.01

^†^Collection period is significant at *P* < 0.01

^#^Collection period is significant at *P* < 0.05

Pigs (n = 12/treatment) were housed individually in metabolism crates and fed diets containing either 0, 2, 4, or 8% D-xylose at 4% of BW. Urine was collected during 3 different periods representing increasing adaptation time to treatment diets.

Threitol was the major metabolite of xylose. Urinary excretion of threitol increased as xylose consumption increased (Trt *P* < 0.0001, linear *P* < 0.0001, quadratic *P* < 0.0001) and, similar to xylose, a treatment by collection period interaction was observed (Trt × Col *P* = 0.0008; Table 7). In pigs fed the xylose-containing diets, the percent of consumed xylose excreted in the urine as threitol increased as dietary xylose concentration increased (Tr *P* < 0.0001, linear *P* < 0.0001, Table 8). This proportion differed across all collection periods with C1 > C3 > C2 (Col *P* < 0.0001).

Xylitol, xylonic acid, and xylulose were minor metabolites of dietary xylose. Urinary excretion of these metabolites also increased as xylose consumption increased (Trt *P* < 0.0001, linear *P* < 0.0001) and significant treatment by collection period interactions were detected (Trt × Col *P* ≤ 0.0021; Table 7). The proportion of consumed xylose excreted as either xylitol, xylonic acid, or xylulose was also impacted by the interaction (Trt × Col *P* = 0.0133; Table 8), but in no instance did it exceed 1.1% of xylose intake.

### Allocation of urine GE

**Fig 3:**
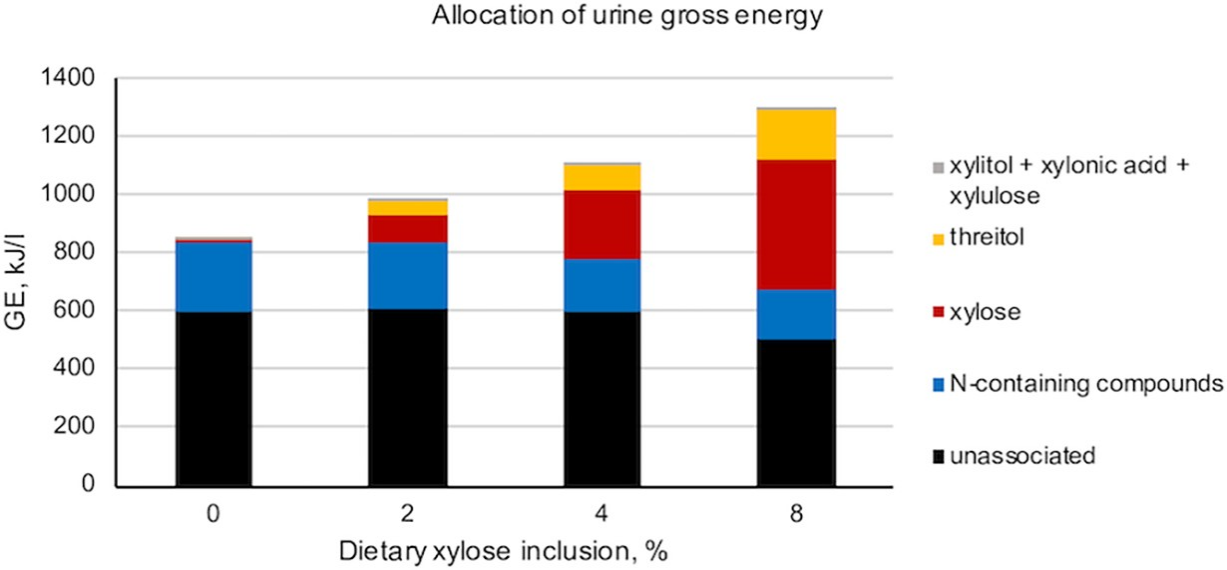
Allocation of urine gross energy (GE) from xylose, metabolites, and nitrogen (N)-containing compounds. Pigs (n = 12/treatment) were fed diets containing either 0, 2, 4, or 8% D-xylose. The presented values are treatment averages across 3 different collection periods. The effect of treatment was significant for all variables (*P* ≤ 0.042). Dietary xylose concentration linearly impacted each variable (linear contrast *P* ≤ 0.012) except the unassociated fraction. Increasing dietary xylose concentration increased the urine GE (kJ/l) contributed from xylose (SEM = 17.4), threitol (SEM = 8.6), and the combination of all lesser metabolites (xylitol, xylonic acid, and xylulose; SEM = 0.3), but decreased the GE contributed from N-containing compounds (SEM = 17.0). The amount of urine GE unexplained by the xylose, its metabolites, or N-containing compounds (SEM = 45.3) was not different among the 0, 2, and 4% treatments (*P* ≥ 0.416) but was lower in the 8% treatment (*P* = 0.042).

Because urine GE was found to significantly increase as dietary xylose concentration increased, it was of interest to determine where the excess urinary energy was coming from. The allocation of urine GE to xylose and its derivatives is presented in Fig 3; values are averaged across collection period. Xylose contributed a large portion of the excess urine energy; the remainder was explained by its metabolite threitol, and to a lesser extent xylitol, xylonic acid, and xylulose. In the 4 and 8% treatments the amount of urine GE coming from N-containing compounds was lower than the control treatment (Trt *P* ≤ 0.0088; Fig 3). Once the differences in energy coming from xylose, its metabolites, and N-containing compounds were accounted for, the remaining unassociated GE was similar among the 0, 2, and 4% treatments, but was lower in the 8% treatment (Trt *P* = 0.0420; Fig 3). The underlying reason for this difference has yet to be determined.

## Discussion

Growing pigs are commonly being fed diets with increasing dietary fiber levels. To help mitigate the negative effects of fiber on growth performance and feed efficiency, exogenous carbohydrases that break down the fiber structure are often supplemented [38,39]. Of the fiber that is found in cereal grains and co-products, a large proportion is hemicellulose and of this, a large fraction is arabinoxylan [40–42], which in turn is primarily composed of the pentose sugars xylose and arabinose. Xylanase releases a portion of the xylose from the xylan backbone [1–3]. This free xylose can then be absorbed in the small intestine [9,43–46] and potentially contribute energy to the pig. However, previous research indicates that xylose is poorly utilized [4], so it is unknown whether xylose would contribute more energy when absorbed as a monosaccharide or through fermentation of arabinoxylan in the hindgut.

Energy is the most expensive component of swine diets [47], so nutritionists formulate diets to precisely balance the concentrations of available energy and nutrients. Therefore, to effectively utilize xylanase to improve the efficiency of pig production, the amount of energy contributed by free xylose must be quantified. While the efficiency and extent of the release of free xylose by xylanase has yet to be determined, the concentration of xylose available for absorption is unlikely to be greater than 4% of diet DM [4]. To our knowledge, only one previous study in pigs has been reported using dietary D-xylose concentrations below 5% [44], and the pig’s ability to adapt and improve its utilization of xylose has yet to be evaluated. This experiment utilized four diets starting with a xylose-free control diet (0%) to which either 2, 4, or 8% xylose was added. Samples were collected in three separate periods over 22 d to evaluate the effects of adaptation time.

### Water balance

Water balance was clearly impacted by xylose consumption in this study. Increasing xylose consumption increased water intake and urine output which agrees with previously reported xylose effects in pigs [9]. We hypothesize that more water was excreted in the urine to balance osmolarity as increasing amounts of xylose and other metabolites were excreted. This hypothesis is supported by the constancy of the specific gravity of urine across all treatments, even though the quantity of solutes excreted per day increased. As adaptation time increased, pigs excreted less xylose in the urine, and water intake and urine output were lower, accordingly. It would be interesting in future studies evaluating xylanase efficacy to measure water consumption and relate this to xylose release.

### Digestibility, fermentation, and diet energy value

Contrary to the results of previous studies [9,43], dietary xylose concentration did not affect total tract digestibility of DM, GE, or CP in biologically relevant ways. Among all treatments and throughout the entire trial, the ATTD of DM ranged from 87.6–89.1% and diet DE was found to be slightly lower in the 2% treatment compared to the other three treatments. Based on previous research [9,43,44] and analysis of a subset of the colon digesta and fecal samples from pigs on this experiment, dietary xylose was assumed to be completely absorbed by the terminal ileum. Because possible microbial fermentation of xylose in the small intestine [9,44] was not evaluated, one potential drawback of this assumption is an overestimation of retained xylose. However, there is empirical evidence that xylose is readily absorbed in the duodenum and proximal jejunum in rats [48,49]. This suggests that the concentration of any unabsorbed xylose would likely be very low in the terminal ileum where microbial populations are more abundant.

The SCFA results from this experiment are consistent with the hypothesis that xylose was almost completely absorbed in the small intestine. Concentrations and molar proportions of SCFA did not differ between the 0 and 8% treatments, indicating that the nutrient composition of substrates fermented in the cecum and colon were similar [50]. In accordance, the pH of cecal digesta did not differ. Because no differences were detected in SCFA concentrations in either the cecum or colon, an explanation for a lower pH in the colon of pigs fed 8% xylose is not directly apparent.

Urine energy concentration linearly increased with increasing dietary xylose concentration, and diet ME decreased with xylose inclusion. Across all collection periods, the 8% treatment consistently provided the lowest diet ME. Although xylose did decrease the ME value of the diet, the relative differences of the 2 and 4% xylose diets compared to the control diet are quite low. For the 2% treatment across C1–3, the ME values were 98%, 97%, and 96% the value of the control diet and for the 4% treatment, the values were 98%, 98%, and 97%, respectively. The 8% xylose diets had the lowest relative ME value at 94%, 95%, and 93% of the control diet during C1–3, respectively. These data clearly show that while the digestibility of the energy in xylose is quite close to that of corn starch, the metabolizability is very different. This is reflected in the composition of the urine, and the relatively high excretion of energy-containing compounds therein. While ME is generally quantitatively closely related to DE in most ingredients [5], it is definitely not in the case of xylose.

Unlike previous studies using dietary xylose concentrations of 10% [43] or 18.7 and 37.4% [51], a significant ADG or feed efficiency response due to dietary xylose inclusion was not detected in pigs on this experiment. This result may be partially explained due to the experimental design of this experiment, including limit feeding, as well as by the relatively small differences in diet ME value among treatments.

### Xylose metabolism

Dietary xylose, adaptation time to the diets (i.e. the effect of collection period), and their interaction significantly impacted the profile of compounds excreted in the urine of pigs. Many of the identified compounds are related to general energy metabolism (ex. malate and citrate), amino acid metabolism (ex. glutaric acid), or are derivatives of microbial metabolism (ex. p-cresol glucuronide and hippuric acid) [52]. The excretion of these compounds was impacted by dietary xylose concentration and indicates that metabolic pathways beyond those directly related to xylose were affected.

Interestingly, sucrose excretion was found to increase with increasing dietary xylose concentrations. Although total urinary sucrose excretion was low (0.03–0.12 g/d), it is uncommon for sucrose to be found in the urine of healthy animals. If sucrose is not hydrolyzed by sucrase at the intestinal brush border, it can be absorbed intact and is usually excreted as such in the urine [53,54]. The fact that urinary sucrose excretion increased due to xylose consumption, even though all diets contained the same amount of sucrose, may support the conclusions of previous studies that xylose inhibits intestinal sucrase activity [55] and reduces the post-prandial glycemic effect of sucrose-containing meals [56,57].

By far, xylose was the most concentrated compound identified in the urine of pigs fed xylose-containing diets. A small amount of xylose was excreted in the urine of the control pigs (0.61 g/d) which is comparable to basal xylose urinary excretion previously reported [44]. As expected, as dietary xylose concentration increased, urinary xylose excretion increased from 6.3 to 40.1 g/d. Adaptation was evident in the 4 and 8% treatments because xylose excretion was significantly lower in C2 and C3 compared to C1. However, in the 8% treatment, xylose excretion was greater in C3 compared to C2.

To be able to compare xylose utilization efficiency on an equivalent basis across all treatments, the proportion of consumed xylose that was excreted in the urine was calculated (Table 8). As xylose consumption increased, the percent excreted in the urine as D-xylose increased from 27.6 to 39.2 to 43.9% in the 2, 4, and 8% xylose treatments, respectively. This was the first indication that the efficiency of xylose utilization decreases proportionally as dietary xylose concentration increases. These value are comparable to previous studies in pigs which reported that 35–53% of absorbed xylose is excreted in the urine [9,43,44].

Previous research has also shown that xylose can be metabolized to other compounds. Radioisotope studies in mammals demonstrated that xylose can be oxidized to xylonic acid [25], CO_2_ [25,58], and be converted to threitol [26,43]. Authors of previous studies have proposed metabolic pathways for some conversions; however, they have generally only been speculated upon for individual reactions [27] or short conversion pathways [25,26]. We have proposed a more comprehensive metabolic pathway to help explain the conversion of xylose to urinary metabolites identified in this study. Metabolic connections with the pentose phosphate pathway (PPP) were shown due to the common assumption that xylose, being a 5-carbon sugar, would be metabolized within the PPP. The connections to the PPP are also likely the means through which xylose can eventually be fully oxidized to CO_2_ and contribute energy to the pig. The proposed pathway was interpreted in conjunction with the cluster analysis of metabolomics data, that indicate which compounds were most similarly influenced by dietary treatment. As discussed below, research in pigs confirms the presence of the enzymes, mostly in the liver and kidneys, which can convert D-xylose into either D-xylonic acid or xylitol.

D-xylose is oxidized to D-xylonic acid by NADP^+^-linked D-xylose dehydrogenase (EC 1.1.1.179) with D-xylonolactone formed as an intermediate. This enzyme and metabolic conversion has been confirmed in pig liver [13–15] with lower activity in the kidney and lens, and even lower activity in the spleen, brain, heart and muscle [16]. The same pathway was demonstrated in guinea pigs using intraperitoneal injections of D-xylose-1-^14^C [25]. The same study then demonstrated that D-xylonic acid-1-^14^C was metabolized much faster than D-xylose and a greater proportion was converted to ^14^CO_2_. This may indicate that D-xylose dehydrogenase could be a rate-limiting step for xylose metabolism. In our study, D-xylonic acid was excreted in the urine at low levels (< 0.6 g/d) but its excretion increased with increasing xylose consumption.

D-xylonic acid can then be converted to D-erythrulose by L-gulonate 3-dehydrogenase (EC 1.1.1.45); this enzyme activity has been confirmed in pig kidney[17]. D-erythrulose can then be reduced by D-erythrulose reductase (EC 1.1.1.162) to D-threitol, the most abundant xylose metabolite identified in the urine in our study. This enzyme (also known as D-threitol:NADP^+^ oxidoreductase) has not been reported specifically in the pig; however, it has been confirmed in the mouse kidney and liver [31] and cattle liver [28,29], with the highest enzyme activity reported in the kidney. If not converted to D-threitol, D-erythrulose can also be phosphorylated to D-erythrulose-4P, which links to the PPP.

In this study, D-threitol excretion increased with increasing xylose consumption—up to 16 g/d in the 8% treatment during C1. If D-threitol is assumed to be a metabolite of dietary xylose, urinary D-threitol represented 11, 13, or 15% of consumed xylose when the 2, 4, or 8% diet was fed, respectively. The identification of D-threitol as the major urine xylose metabolite corroborates previous studies in pigs [43] and humans [26] which both reported that 10–11% of ingested xylose was excreted as threitol. Pitkänen [26] specified that in the first 5 h after ingestion only 4% of the oral xylose dose was excreted as threitol; when the collection was extended to 24 h, the proportion increased to 10.6%.

In the literature, the ME value of diets is consistently reduced by xylose inclusion [4,9], but it is likely that in addition to excreted xylose, threitol also significantly accounts for the reduction in ME. This was apparent in our study and likely was an unidentified causative factor in the studies by Verstegen et al. [43] and Schutte et al. [9]. Furthermore, threitol likely explains the unidentified compound in guinea pig urine that contained 18% of an intraperitoneal injection of D-xylose-1-^14^C [25].

D-xylose can also be oxidized to xylitol via aldose/aldehyde reductase (EC 1.1.1.21). This conversion has been confirmed in the cortex of pig kidney [18,59], but the reaction seems to occur at a slow rate [18]. The enzyme has also been identified in the lens and skeletal muscle of pigs [19]. Xylitol was the second most abundantly excreted metabolite of xylose, but at most, only 0.41 g/d were excreted. In all xylose-containing treatments, xylitol excretion was the greatest during C3, potentially indicating a slow metabolic upregulation of aldose/aldehyde reductase as pigs adapted to the diets. Xylitol was also identified as a metabolite in humans infused with D-xylose-1-^14^C, but represented < 0.1% of the recovered ^14^C [58].

Xylitol can be converted to either the D- or L-isomer of xylulose. In this study, D-xylulose excretion was found to increase with increasing xylose consumption. Xylitol dehydrogenase (EC 1.1.1.14) oxidizes xylitol to D-xylulose and this enzyme is distributed in many mammalian tissues of which, the liver is the main organ [23]. Finally, in the liver and kidneys, D-xylulose can be phosphorylated by xylulokinase (EC 2.7.1.17) to D-xylulose-5P[35], an intermediate in the PPP. Xylitol, D-xylulose, and D-xylonic acid were determined to be minor metabolites of dietary xylose, and together represented 0.58–1.03% of consumed xylose.

Taking into account xylose that was either excreted in the urine directly or excreted as threitol or a minor metabolite, the proportion of consumed xylose that was retained in the body ranged from 27–63%. Increased dietary xylose concentration decreased the retention efficiency across all collection periods. However, a larger proportion of xylose was retained during C2 and C3, i.e. after pigs had been adapted to the treatment diets for at least 11 d. This indicates that pigs are able to adapt metabolically to utilize xylose more efficiently. Further research is necessary to determine how this occurs, which mechanisms may be up or down regulated, and the final metabolic fate of retained xylose in pigs.

Dietary xylose concentration clearly impacts its retention efficiency. This is a consistent finding across studies in pigs and other species, as reviewed earlier this year by Huntley and Patience[4]. Considering these data in the context of commercial pig production, it is unlikely that exogenous xylanase supplementation would release free D-xylose in the small intestine at concentrations > 4% of the total diet DM. Based on the results of this experiment, only about 50–63% of the xylose released would be retained and could potentially contribute energy to the pig through oxidation. Experiments must still be conducted to directly compare the net energetic value of xylose through metabolic oxidation verses fermentation. To speculate, based on the xylose retention efficiency measured in pigs fed 4% xylose in this study (51%), and using the metabolic energy efficiencies of xylose reported by Yule and Fuller [44] and reported energetic efficiencies of fiber fermentation in the pig [60–62], it is estimated that xylose would provide approximately 15% less energy if absorbed in the small intestine vs. through fermentation.

Further research is needed to determine how much free xylose is released from arabinoxylan in the small intestine of pigs due to xylanase supplementation. Furthermore, it would be valuable to examine the liver and kidney for enzymes that may be differentially expressed and regulated in pigs consuming xylose. This will help determine how adaptation to xylose-containing diets occurs, and how other metabolic processes may be affected. Understanding potential effects on other metabolic processes may also help to explain the decreased proportion of urine GE not associated with xylose, its metabolites, or N-containing compounds in pigs fed 8% xylose diets.

## Conclusions

In conclusion, pigs can metabolize xylose, but with considerably lower efficiency than glucose. Furthermore, pigs can adapt over time to utilize xylose more efficiently, as evidenced by the decline in the portion of dietary xylose excreted in the urine directly or as its metabolites. Xylose retention decreased from 60% to 47% to 41% when pigs were fed diets containing 2, 4, or 8% xylose, respectively. Thus, increasing dietary xylose concentration decreases diet ME. A comprehensive pathway for xylose metabolism in the pig is proposed and D-threitol was confirmed as the major urinary metabolite of xylose. Xylitol, D-xylonic acid, and D-xylulose were identified as minor metabolites.

This experiment provides novel data regarding xylose metabolism when fed at relevant concentrations for typical swine diets. To fully understand these data in the context of practical swine nutrition, further research is needed to determine how much free xylose is released from arabinoxylan with xylanase supplementation. Because of the importance and cost of energy in swine diets, assigning an energy release value to xylanase is of great interest. These data indicate that xylose’s energy contribution to the pig will depend on the amount of xylose released and the length of adaptation to the diet.

## Supporting information

S1 Data Data file. (XLSX)
